# Oxidative Stress in Non-Dialysis-Dependent Chronic Kidney Disease Patients

**DOI:** 10.3390/ijerph18157806

**Published:** 2021-07-23

**Authors:** Patricia Tomás-Simó, Luis D’Marco, María Romero-Parra, Mari Carmen Tormos-Muñoz, Guillermo Sáez, Isidro Torregrosa, Nuria Estañ-Capell, Alfonso Miguel, José Luis Gorriz, María Jesús Puchades

**Affiliations:** 1 Nephrology Department, Hospital Clínico Universitario, INCLIVA, Universidad de Valencia, 46010 Valencia, Spain; p.tomas.simo@gmail.com (P.T.-S.); Romero.parra.maria@gmail.com (M.R.-P.); isist67@gmail.com (I.T.); juan.a.miguel@uv.es (A.M.); jlgorriz@gmail.com (J.L.G.); 2 Service of Clinical Analysis, Department of Biochemistry and Molecular Biology, Facultad de Medicina y Odontología-INCLIVA, Hospital Universitario Dr. Peset, FISABIO, Universidad de Valencia, 46010 Valencia, Spain; m.carmen.tormos@uv.es (M.C.T.-M.); guillermo.saez@uv.es (G.S.); estany_nur@gva.es (N.E.-C.)

**Keywords:** oxidative stress, chronic kidney disease, cardiovascular disease

## Abstract

Background: Cardiovascular complications are the leading cause of morbidity and mortality at any stage of chronic kidney disease (CKD). Moreover, the high rate of cardiovascular mortality observed in these patients is associated with an accelerated atherosclerosis process that likely starts at the early stages of CKD. Thus, traditional and non-traditional or uremic-related factors represent a link between CKD and cardiovascular risk. Among non-conventional risk factors, particular focus has been placed on anaemia, mineral and bone disorders, inflammation, malnutrition and oxidative stress and, in this regard, connections have been reported between oxidative stress and cardiovascular disease in dialysis patients. Methods: We evaluated the oxidation process in different molecular lines (proteins, lipids and genetic material) in 155 non-dialysis patients at different stages of CKD and 45 healthy controls. To assess oxidative stress status, we analyzed oxidized glutathione (GSSG), reduced glutathione (GSH) and the oxidized/reduced glutathione ratio (GSSG/GSH) and other oxidation indicators, including malondialdehyde (MDA) and 8-oxo-2’-deoxyguanosine (8-oxo-dG). Results: An active grade of oxidative stress was found from the early stages of CKD onwards, which affected all of the molecular lines studied. We observed a heightened oxidative state (indicated by a higher level of oxidized molecules together with decreased levels of antioxidant molecules) as kidney function declined. Furthermore, oxidative stress-related alterations were significantly greater in CKD patients than in the control group. Conclusions: CKD patients exhibit significantly higher oxidative stress than healthy individuals, and these alterations intensify as eGFR declines, showing significant differences between CKD stages. Thus, future research is warranted to provide clearer results in this area.

## 1. Introduction

Patients affected by renal disease are extremely vulnerable to cardiovascular disease (CVD). Indeed, cardiovascular complications are the leading cause of morbidity and mortality at any stage of chronic kidney disease (CKD), with annual mortality rates 10–20-fold higher than in the general population even after adjusting for age, sex, ethnicity and presence of diabetes mellitus [1]. Moreover, the high rate of cardiovascular mortality observed in these patients are related to an accelerated atherosclerosis process that probably starts in early stages of the disease. Although conventional cardiovascular risk factors are highly prevalent in the renal-affected population from the initial stages of CKD, this alone cannot explain the high cardiovascular risk observed. Beyond traditional CVD risk factors in the general population, such as hypertension, diabetes, dyslipidemia and tobacco, more specific or uremic-related factors may play a key role in the pathogenesis of CVD in CKD patients [2,3,4].

Among these non-traditional risk factors, there has been a particular focus on anaemia, mineral and bone disorders, insulin resistance, inflammation, malnutrition and oxidative stress [4,5,6]. Among these, it can be argued that the increased production/accumulation of oxidative stress is a link and unifying factor between CKD and increased cardiovascular risk, since this pathological environment disturbs the excretory function of the nephron and other regulatory mechanisms, such as tubular glomerular feedback, myogenic reflex in the arteriole, and the renin–angiotensin–aldosterone system [7]. As a result, these imbalances lead to an additional increase in oxidative stress and CKD progression [8].

**Figure 1:**
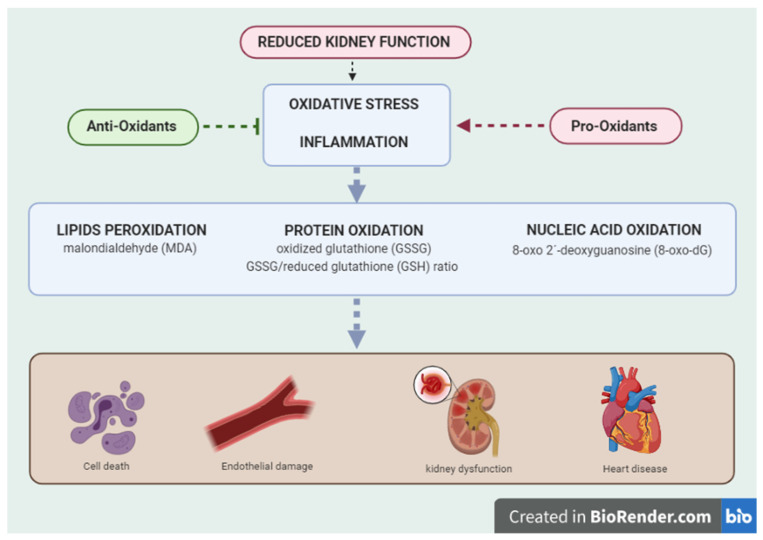
**Oxidative stress and inflammation as key factors in the development of kidney and cardiovascular damage**. The decline in kidney function is associated with an increase in oxidative stress and meta-inflammation. When this process become chronic, important metabolic changes and alterations in vascular renal hemodynamic stimulated chronic renal failure and cardiac dysfunction.

There is experimental evidence that implicates reactive oxygen species (ROS) as primary mediators in the pathogenesis of kidney damage caused by ischemic, toxic processes and antibody–antigen reactions at the glomerular and tubular level. Oxidative stress also generates subclinical inflammation which induces endothelial dysfunction and vascular aging, reducing the availability of nitric oxide [7,8]. This process, known as meta-inflammation, is defined as a chronic, low-grade inflammatory state induced by metabolic changes and is linked to CKD and chronic heart failure [9]. Recent evidence has identified key mediators and pathways that link metabolic disturbances and chronic inflammation with target organ injury. These include mitochondrial dysfunction and oxidative stress. Oxidative stress markers, such as malondialdehyde (MAD), 8-oxo-7,8-dihydro-20-deoxyguanosine, advanced oxidation protein products (AOPPs) and carbamylated proteins, as well as asymmetric dimethylarginine (ADMA) and oxidized lipoprotein particles have been shown to overproduce and accumulate in CKD as renal dysfunction progress (Figure 1) [7,8,10,11,12].

In this regard, connections have been reported between oxidative stress and CVD in dialysis patients [13,14,15], whereas evidence in the non-dialysis population is still inconclusive. Most studies available to date are focused on small oxidative biomarkers [16], and often on populations with heterogeneous characteristics, which makes gaining an overview of this disorder complicated [17]. The aim of this study is to evaluate the oxidation process in different molecular lines (proteins, lipids and genetic material) in patients at different stages of CKD.

## 2. Methods

This cross-sectional study was conducted over 30 months in 155 CKD non-dialysis-dependent (CKD-NDD) patients from the outpatient clinic of the nephrology department at the Hospital Clínico Universitario of Valencia. The study was approved by the research ethics committee of the hospital, and all patients signed consent forms in accordance with the Declaration of Helsinki.

### 2.1. Study Inclusion Criteria

Patients aged between 18-80 years, with previous CKD diagnosis at least 12 months before study initiation and clinical stability during the last 6 months were included.

### 2.2. Exclusion Criteria

Patients with active neoplasia or inflammatory, present or recurrent infectious disease, having received intravenous iron in the last six months or blood transfusion in the 15 days prior to blood sample extraction were excluded.

### 2.3. Population

Of the 157 patients screened, two were excluded due to active infection. Finally, 155 CKD-NDD patients at stages 3–5 were included in the study, along with a control group of 45 healthy subjects with similar demographic data.

All patients attended our outpatient clinic at baseline and we reviewed the medical records of these patients, recording their demographic data and clinical characteristics: etiology of kidney disease, history of smoking, hypertension, dyslipidemia, diabetes mellitus, ischemic heart disease (defined by history of myocardial infarction, angina, or positive imaging test for myocardial ischemia), arrhythmia, heart failure, stroke, or peripheral arterial disease.

Blood samples were collected after 12 h of fasting, for haematology and biochemical screening, including urea, creatinine, lipid and protein panel, albumin and phospho-calcium metabolism. Renal function was measured by the estimated glomerular filtration rate (eGFR) using the CKD-EPI formula, and patients were classified according to CKD stage following KDIGO guidelines [18].

### 2.4. Oxidative Stress Study

To determine oxidative stress parameters in each patient, 14 mL of blood was withdrawn in a tube with EDTA as an anticoagulant. These samples were immediately centrifuged to separate plasma. Mononuclear cells were isolated by Ficoll–Hypaque centrifugation, followed by three saline washes. Mononuclear cells were resuspended in RPMI 1460 medium (Sigma) and lysed with RNA/DNA stabilization Reagent for Blood/Bone marrow mar lysis buffer (Roche) for DNA extraction were then stored at −80 °C until use. Nuclear DNA was isolated following the Gupta method, with the modification described by Muniz et al. [19] in which alcohol isoamyl chloroform (24:1) is used instead of phenol for protein removal.

The DNA obtained was quantified by spectrophotometry, diluting the sample 1/100 in TE buffer (10 mM Tris-Cl, 1 mM EDTA) and measuring the absorbance of the dilution at two different wavelengths:

- 260 nm: wavelength of peak nucleic acid absorption.
- 280 nm: wavelength of maximum protein absorption.

The relationship between the readings at 260 nm and at 280 nm helps us determine the purity of the extraction. If this ratio is greater than 1.8, the DNA purity is considered acceptable; if not, the sample is discarded.

To study lipid peroxidation, malondialdehyde (MDA) was determined in isolated mononuclear cells by high resolution liquid chromatography, and the protein concentration in each sample was measured by Lowry’s method to reference these parameters [20].

Pro-oxidation analysis was studied by determining oxidized glutathione (GSSG), reduced glutathione (GSH) and the relationship between GSSG/GSH by high-performance liquid chromatography in mononuclear cells following the methods described by Ursini and Navarro [21].

Lastly, isolation and the analysis of the modified base 8-oxo 2′-deoxyguanosine (8-oxo-dG) was performed by HPLC-EC in previously isolated and digested nuclear DNA following the conditions described by Frenkel et al. [22].

### 2.5. Statistical Analysis

Variables were expressed as mean ± standard deviation and as median and interquartile range for variables with non-normal distribution (Shapiro–Wilk test). Next, logarithmic transformation was performed. For comparison of two independent variables, T student with Levene test was used for equality of variances. To compare qualitative variables, chi square with the Yates correction was performed; and in case of less than five observations we used Fischer’s test. To study different correlations between variables, Pearson’s bivariate correlation was performed. There were no missing data among the included patients and significance was set at *p* ≤ 0.05.

One-way ANOVA was performed for comparison analysis between the five groups of all the oxidative stress parameters studied. The percentiles of the oxidative stress parameters in the control group were also calculated. The 90th percentile of the oxidized variables was considered the upper level of normality and lower level of normality was the 10th percentile of antioxidant variables. MedCalc version 15.4 and SPSS for Windows version 19 were used for statistical analysis.

## 3. Results

The final sample of the study consisted of 155 CKD-NDD patients at different stages of CKD, 51.6% of whom were women with an average age of 68.64 years; 48.4% were men with an average age of 68.09 years. The baseline characteristics of these patients, demographic data and primary cause of kidney disease are summarized in Table 1 stratified by CKD stage.

The prevalence of cardiovascular risk factors observed in the study are shown in Table 2. We grouped CKD etiology into the following categories: vascular, interstitial, diabetic and polycystic kidney disease, glomerulonephritis, unknown causes and other.

Most patients showed a high percentage of conventional cardiovascular risk factors across the different stages of CKD. Moreover, hypertension and dyslipidemia were present in more than 90% of patients, even in earlier stages of CKD. Notably, no significant differences were found between CKD groups, except for homocysteine between G3A and all remaining groups (*p* < 0.05). The prevalence of CVD distributed by CKD stage is described in Table 3.

More than 50% of patients had already presented with heart disease since initial stages of CKD. All CKD groups showed similar cardiovascular comorbidities. Only coronary heart disease showed a significant increase when compared to the rest of CKD stages (*p* = 0.04). No other significant between-group differences were detected in the remaining comorbidities (Table 3). Patient baseline biochemical characteristics are shown in Table 4.

### 3.1. Oxidative Stress

Oxidative stress analysis results in CKD-NDD patients and controls are shown in Table 5. CKD patients (in all stages) presented significantly higher values in oxidative stress parameters than the control group: (lipid peroxidation (MDA), *p* = 0.009; thiol oxidation-derived products (GSSG, GSSG/GSH, *p* = 0.08). When comparing the means obtained from controls against renal-affected patients, we found a statistically significant difference (*p* < 0.001) in all oxidative stress parameters studied.

**Figure 2:**
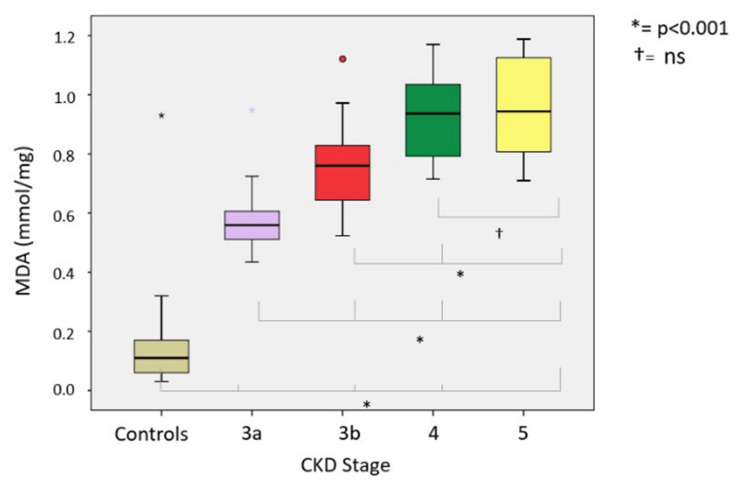
MDA levels in different stages of CKD.

**Figure 3:**
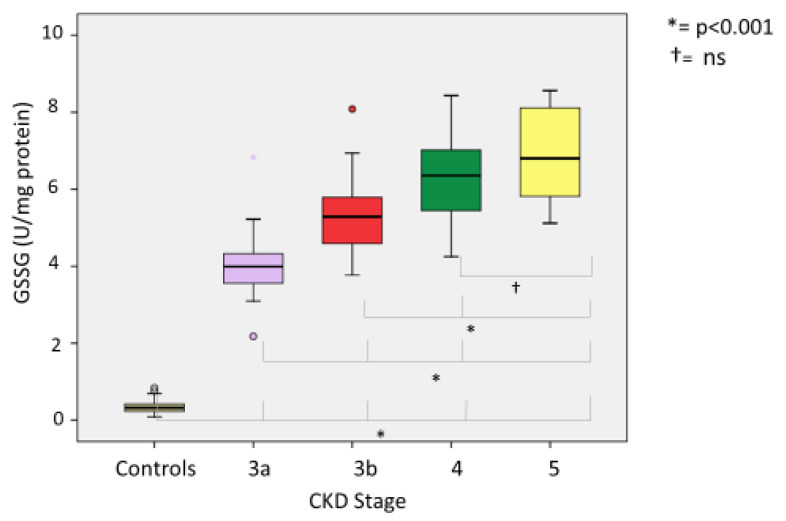
GSSG levels in different stages of CKD.

**Figure 4:**
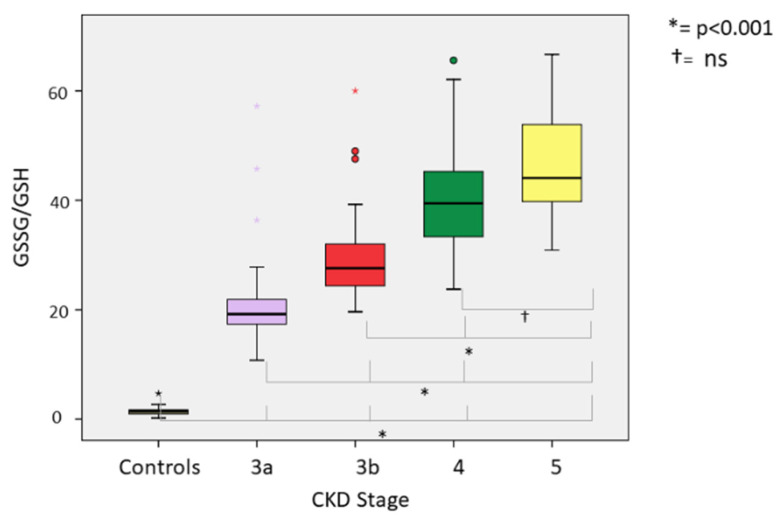
GSSG/GSH ratio in different stages of CKD.

**Figure 5:**
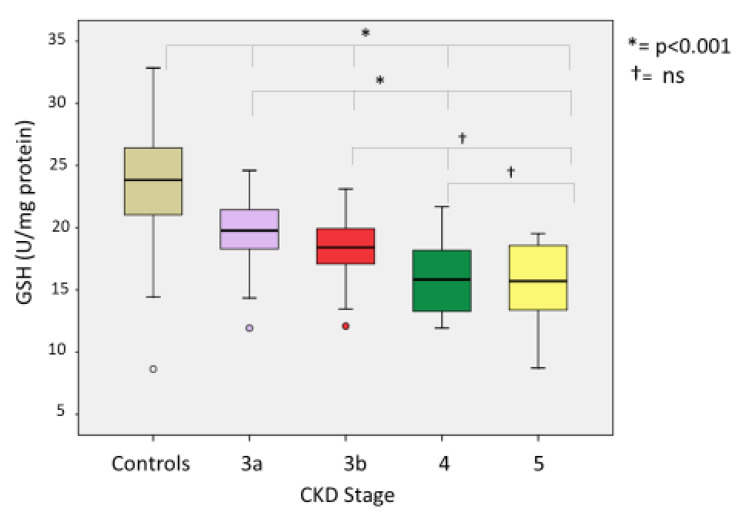
GSH in different stages of CKD.

Comparing oxidative stress parameters, we found significant differences in CKD groups compared with the controls. Our analysis of the differences between CKD stages (3A, 3B, 4 and 5) revealed the following: (a) At lipid peroxidation level (MDA) differences were statistically significant (*p* < 0.001) between all groups except between CKD G4 and G5-NDD (*p* = 1000) (Figure 2). (b) Among thiol oxidation-derived products (GSSG, GSSG/GSH), significant differences were also found (*p* < 0.001) between all stages of CKD (Figure 3); however, the GSSG/GSH ratio between CKD G4 and G5-NDD was not significant (*p* = 0.080). (c) Statistically significant variations (*p* < 0.001) in levels of 8-oxo-dG—a molecule derived from oxidative damage of nuclear genetic material—were identified between all stages of CKD, although no association was observed between CKD G4 and G5-NDD (*p* = 1.000) (Figure 4). (d) Regarding the antioxidant parameter (GSH), the only significant difference found was between G3A and G3B (*p* < 0.001), compared to other stages (*p* = 0.232 between G3A and G3B; *p* =1.000 between G4 and G5-NDD) (Figure 5).

The highest mean values of all oxidative parameters were observed in CKD stage G5. Of note, CKD stages G4 and G5 showed no statistically significant differences in oxidative stress levels. Nonetheless, for oxidative parameters, the mean values in patients with CKD were higher than the 90th percentile of the control group. Whether the CKD group was considered as a whole or separately across CKD stages was observed. In contrast, mean levels of antioxidant parameters were lower in the CKD population than in the control group. On stratifying CKD groups by stages, the mean values of all oxidative parameters were above the 90th percentile of the control group at all stages. The same occurred with the oxidative stress parameters, with the exception of CKD G3A, whose mean value was higher than the 10th percentile of the control group.

**Figure 6:**
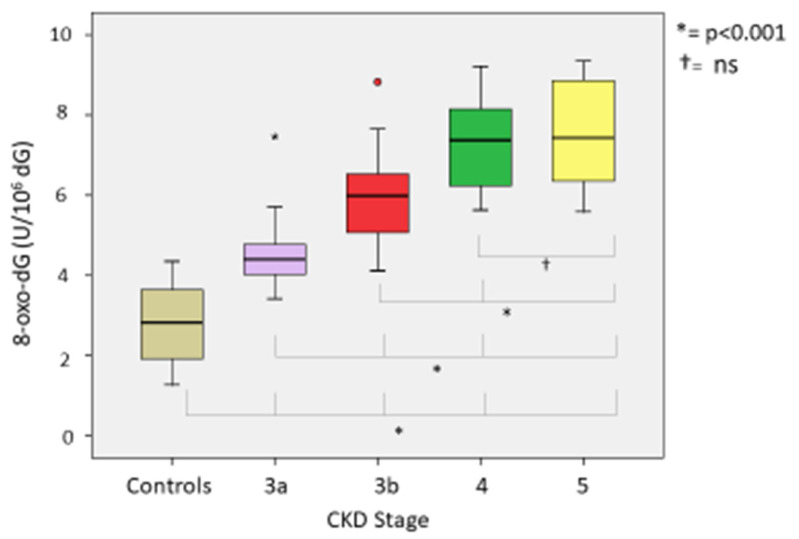
8-oxo-dG in different stages of CKD.

We next determined the percentage of patients at each stage with values above or below the calculated percentiles, with the following findings. All patients across the four groups were found to have MDA levels above 0.195 nmol/mg protein (90th percentile of control group); except for one patient in G3A, 99% of patients had higher GSSG values at 0.646 U/mg protein; 100% of GSSG/GSH ratios in all study groups were above 2.218; the percentage of patients with measurements higher than 4.19 U/10^6^ dG of 8-oxo-dG increased across stages: 62.1% in G3A, 78.3% in G3B, 87.24% in G4 and 91.67% in G5 (Figure 6); finally, only 35.1% of patients in G3A presented higher GSH levels of 19 U/mg protein, while the remaining patients always presented lower values.

### 3.2. Correlation of Oxidant and Antioxidant Parameters

Analysis of the correlation between oxidative parameters showed a positive correlation between all the oxidizing molecules (8-oxo-dG, GSSG, GSSG/GSH and MDA); however, a negative correlation with antioxidant molecule (GSH) (*p* < 0.001) was found. 

### 3.3. Correlation between Glomerular Filtration and Oxidative and Antioxidant Parameters

We evaluated a possible relationship between eGFR and different oxidative stress parameters. All oxidative stress parameters were correlated with eGFR (CKD-EPI formula) levels (*p* < 0.001), revealing a negative relationship with all the oxidizing molecules (R between −0.684 and −0.653) and a positive one with the antioxidant (R = 0.441) parameters.

### 3.4. Correlation between Oxidative Stress and Diabetes

Interestingly, we found no statistical differences between diabetic kidney disease-affected patients and those without this condition. A total of 74 diabetic patients were compared to 81 non-diabetic patients and none of the mean oxidative stress parameters showed significant differences (*p* = 0.056–0.147).

## 4. Discussion

The main finding in this study was an active grade of oxidative stress in CKD-NDD patients, with onset at early stages of the disease, involving all molecular lines studied. We observed a heightened oxidative state, determined by higher levels of oxidized molecules together with decreased levels of antioxidant molecules, as kidney function worsened. Furthermore, oxidative stress-related disturbances were significantly greater in CKD patients than the control group.

Evaluating the role of oxidative stress in the pathogenesis of different diseases is complicated principally by the short half-life of free radicals and the difficulty of directly detecting these species in biological samples. This highlights the need to study biomarkers that reflect changes taking place at a cellular level and at the matrix of macromolecules, including sugars, proteins, deoxyribonucleic acid and lipids [23].

Increasing evidence suggests that oxidative stress may be pivotal in the development of cardiovascular complications in CKD patients, playing an important role in the pathogenesis of vascular injury and in the progression of atherosclerosis disease and, therefore, linked to endothelial dysfunction [24]. Oxidative stress has also been implicated in the progression of kidney damage along with angiotensin II, aldosterone and metabolic acidosis [25], and has been suggested to participate in non-hemodynamic factors, directly through glomerular damage and renal ischemia or indirectly associated with inflammation, hypertension and endothelial dysfunction [26]. The pathological link between oxidative stress, inflammation and CKD progression is, therefore, based on an initial kidney injury arising from intra- and extracellular activity of the ROS and the resulting inflammatory response [27]. To address this issue, studies have described a direct renal damage as resulting from amino acid oxidation, resulting in loss of important functional properties of proteins [28]; from lipid peroxidation of cell membranes [29,30], with a consequent decrease in cellular membrane viability; from cleavage of kidney DNA leading to deleterious mutations and alteration of cellular function [31]. ROS-mediated damage in the nephron elicits an inflammatory response, which normally serves as a protective and restorative mechanism, stimulating additional free radical formation. When this process is prolonged over time (chronic inflammation), extensive and irreversible tissue damage may affect kidney functions [32].

Our results are in agreement with previously published studies [33]. At lipid peroxidation level, Atamer et al. demonstrated that the levels of MDA, lipoprotein (a) and homocysteine were significantly higher in CKD patients (n = 60) than in controls, while activity of the enzyme paraoxonase 1 (PON-1) (a component of HDL, with protective role against atherosclerosis by preventing lipoprotein oxidation) was lower [34]. Other research carried out by Puchades et al. in 32 CKD G4 patients (mean eGFR, 22.1 ± 1.08 mL/min) showed significant differences in MDA levels between CKD patients and control groups [35]. Similarly, we found significantly high levels of MDA in CKD patients compared to the control group (0.79 ± 0.2 vs. 0.11 ± 0.05; *p* < 0.001); moreover, we observed that MDA levels increased at worse stages of CKD. Furthermore, all patients presented higher levels of MDA than the 90th percentile of the control group.

Given the paucity of studies in the dialysis population analyzing the oxidative damage of genetic material in patients with renal disease, we explored this issue using the molecule 8-oxo-2′-deoxyguanosine (8-oxo-dG) in CKD-NDD patients [36,37]. Tarng et al. showed that 8-oxo-dG content in nuclear DNA provides a reliable measure of oxidative damage of genetic material in peripheral leukocytes of patients with reduced eGFR (<20 mL/min/m^2^) and chronic haemodialysis [17,38]. Rangel-López et al. published a study in pre-dialysis (mean eGFR 25 mL/min/m²), peritoneal dialysis and haemodialysis patients, which examined the degree of damage to genetic material and its relationship with markers of oxidative stress and inflammation [39]. They compared the values obtained with a control group of 61 patients (48 young controls: mean age, 33.9 ± 5.8 years; and 13 older controls: mean age, 49.1 ± 5.7 years), observing that 8-oxo-dG levels were higher in the CKD groups (n = 91) compared to controls. Similarly, 8-oxo-dG levels were higher in the CKD G5-NDD group than in the dialysis group, suggesting that the elimination of uremic toxins during dialysis could reduce oxidative damage to nuclear genetic material. In this regard, our study has shown that 8-oxo-dG levels were higher in CKD-NDD patients than in controls. Nonetheless, analyzing by CKD stages, we observed as a novel finding that an increase in oxidative damage is present at the earliest stages of CKD which rose inversely with declining kidney function, reaching peak levels in CKD-NDD G5 patients. Further, we observed significant differences between all stages of CKD except G4 and G5-NDD.

The generation and accumulation of uremic toxins favors free radical production and, therefore, molecular oxidation with the increase of oxidized substances, which has an impact on erythropoiesis. Hence, reduced mitochondrial DNA copy number, reduced energy production, and higher levels of stress markers contribute to this condition in CKD. Moreover, the decrease in the ability to synthesize erythropoietin due to kidney disfunction causes the appearance of anemia [6]. On the other hand, the significant decrease in antioxidant capacity with the decline in kidney function is relevant. Of interest, has been suggested that iron, when supplied in excess, leads to oxidative stress in the mitochondria triggering the initiation of the Fenton reaction and promote the formation of ROS with adverse consequences. Our results failed to provide association regarding this issue.

Protein oxidation is assessed by measuring oxidized glutathione (GSSG: glutathione disulfide) and the ratio of oxidized glutathione to reduced glutathione (GSSG/GSH), an indicator of oxidative status of the cell and cellular toxicity [40]. We also evaluated reduced glutathione (GSH) as an antioxidant molecule, to complete our analysis of the redox cycle of glutathione. This cycle has been studied as part of oxidative stress in CKD patients. Ceballos et al. conducted research in 223 uremic patients: 185 with mild-moderate CKD and 48 patients on chronic dialysis programs (peritoneal dialysis or haemodialysis) were compared with a control group [41]. GSSG-Rd and GSH-Px activity was found to significantly increase in pre-dialysis stages, while total GSH level decreased; however, no significant differences were observed in GSSG levels. They concluded that alterations in antioxidant systems gradually increased with degree of kidney failure, being higher in dialysis patients and especially in those on chronic haemodialysis, in whom there was almost complete suppression of GSH activity. Importantly, a positive correlation between creatinine clearance and plasma levels of GSH-Px and GSH was found. Hence, disturbances in antioxidant systems begin at earlier stages of chronic uraemia and are exacerbated by dialysis treatments. Similarly, Annuk et al. [42] subsequently studied the relationship between different oxidative stress markers and degree of kidney disease in 38 patients with CKD in pre-dialysis stages and 61 healthy controls. They found that GSSG and GSSG/GSH levels were increased, GSH level decreased and resistance of the lipoprotein fraction to oxidation was severely reduced compared to healthy controls. Serum levels of creatinine and urea were correlated with GSSG and GSSG/GSH levels in CKD patients. They concluded that not only do CKD patients maintain an active oxidative state compared to healthy controls, but also that glutathione redox status and GSSG levels are related to the degree of kidney failure. Our previous reports also showed higher GSSG/GSH levels in CKD-NDD and dialysis patients than in controls [35,43]. As previously commented, CKD patients have an increased risk for CVD [44]; nonetheless, this risk has not been significantly reduced with the current available therapies. Oxidative stress is among the non-standard factors that can influence residual kidney risk [42], making it vital to enhance our understanding of the mechanisms involved in the pathogenesis of oxidative stress and kidney injury. Moreover, new therapeutic targets are under investigation to improve oxidative status in CKD patients and many of these complement currently available treatments [45]. Several strategies have recently been developed, such as supplementation with vitamin E and zinc (antioxidant molecules) in diabetic kidney disease, which has produced beneficial effects on diabetic nephropathy by decreasing microalbuminuria, but without slowing CKD progression or avoiding dialysis initiation [46,47]. Another report, focusing on antioxidant protection at the renal level (the BEACON trial), studied bardoxolone methyl, an inducer of Nrf2 (erythroid-derived nuclear factor-2 similar to factor 2) and inhibitor of NF-κB (nuclear factor kappa- activated B-cell light chain enhancer). The authors reported that Bardoxolone methyl preserves kidney function and may delay the progression of CKD in patients with type 2 diabetes mellitus [48]. Selonsertib, a potent selective inhibitor of apoptosis signal regulating kinase 1, has been postulated to prevent apoptosis caused by increased oxidative stress in glomerular and tubular cells. A recently published randomized trial used selonsertib vs. placebo to evaluate efficacy, safety, and tolerability in participants with diabetic kidney disease [49]. Although the trial did not meet its primary endpoint, exploratory post hoc analyses suggest that selonsertib may slow diabetic kidney disease progression. Of note, the relationship between type 2 diabetes mellitus and oxidative stress is well established, yet our results did not show this correlation in CKD patients. Both processes are known to increase oxidative stress with greater or lesser intensity. The lack of difference between CKD patients with or without type 2 diabetes mellitus could be related to an oxidation status threshold point, above which no further increases may take place or can be detected.

The decrease in antioxidant systems, together with an increase in oxidative stress markers that accompany the pathogenesis of different diseases, has led to speculation, not yet fully substantiated, about the effectiveness of administrating exogenous antioxidants.

In some clinical trials, antioxidants showed either no or even a negative effect in terms of increased mortality, with conflicting data. Various aspects and mechanisms may contribute to the failure of antioxidant use as therapeutic agents. These were recently reviewed by Schmidt, HH et al. [50]. Appropriate administration of antioxidants must take into account several aspects, mainly relating to differing mechanisms and sites of endogenous or exogenous ROS production, cell type and subcellular compartment affected, and also the type of species being formed: superoxide, hydrogen peroxide or hydroxyl radical. Furthermore, certain recommendations of use must also be followed, mainly in CKD patients; its administration should be limited to situations of acute or sustained ROS production. Finally, strategies have been suggested to induce endogenous antioxidant production rather than directly administering a specific antioxidant. It has been demonstrated that maintaining redox equilibrium with low levels of ROS may have substantial beneficial effects which prove particularly useful for cell homeostasis. Among the limitations of our study are the small sample size and unbalanced gender ratio in the control group, and lack of patient data on ethnicity, physical activity, body mass index and nutritional status. Likewise, analysis of other antioxidants, such as thioredoxin, Glutathione peroxidase (GPX), or catalase, or of inflammatory biomarkers, such as hsPCR or interleukin were beyond the scope of the study. Moreover, we only evaluated systemic markers of oxidative stress, without including other biological samples or tissues and albuminuria/proteinuria. 

## 5. Conclusions

In conclusion, CKD patients show significantly higher oxidative stress levels than healthy individuals, and these changes are intensified as eGFR declines, showing marked differences between CKD stages. Moreover, differences in oxidative stress parameters affected the three molecular lines studied equally. The process begins in the early stages of renal disease and increases along the course of deterioration, while antioxidant capacity decreases. Although future research is warranted to provide clearer results in this area, our findings are important, particularly in the pre-dialysis population, in whom current therapies are unsuccessful in slowing down CKD progression and reducing cardiovascular risk.

## Figures and Tables

**Table 1:** Baseline characteristics, demographic data and primary cause of CKD.

	G3A	G3B	G4	G5-NDD	CONTROLS
eGFR, mL/min/1.73 m^2^	(60–45)	(45–30)	(30–15)	(<15)	(≥90)
Number of patients	37	47	47	24	45
Male (%)	67.6	74.5	57.4	66.7	51.1
Age, years *	65.49 (31–80)	68.62 (44–80)	69.26 (42–80)	68.79 (45–80)	67.8 (33–80)
Etiology (%)					
Nephroangiosclerosis	41.2	37.9	29.9	52.1	
Diabetic kidney disease	25.5	16.4	22.3	19.4	
Interstitial Nephritis	5.8	15.3	15.7	8.1	
Obstructive nephropathy	5.9	2.2	4.3	--	
Polycystic kidney disease	8.8	8.7	8.5	4.3	
Glomerulonephritis	5.9	8.7	10.6	6.1	
Others/Unknown etiology	6.8	10.9	8.6	9.9	

* Mean + interquartile range (IQR). No significant differences between groups (sex, age and primary renal disease) were observed.

**Table 2:** Prevalence of global cardiovascular risk factors according to CKD stages.

Cardiovascular Risk Factors (%)	G3A	G3B	G4	G5-NDD	TOTAL
Active smoker	29.4	19.6	24.4	17.4	22.7
Hypertension	91.2	93.5	93.6	91.3	92.4
Hypercholesterolemia	79.4	89.1	91.5	95.7	88.9
Hypertriglyceridemia	23.5	23.9	21.3	30.4	24.8
Type 2 Diabetes Mellitus	54.1	44.7	48.9	41.7	47.4
Hyperhomocisteinemia	35.13	65.95	80.85	91.66	68.3

**Table 3:** Prevalence of cardiovascular disease, global and by stages.

CARDIOVASCULAR DISEASE (%)	G3A	G3B	G4	G5-NDD	TOTAL
Coronary heart disease	52.9	63	65.2	76.6	64.4
Atrial fibrillation	8.8	10.9	17	17.4	13.5
Heart failure	20.6	26.1	36.2	26.1	27.3
Cerebrovascular disease	8.8	13	6.4	4.3	8.1
Peripheral artery disease	23.5	21.7	21.3	39.1	26.4

**Table 4:** Basic biochemical profile of the renal population by stages.

Biochemical/CKD	G3A	G3B	G4	G5-NDD
eGFR (ml/min/1.73 m^2^) *	50.55 ± 3.97 49.01–52.09	37.28 ± 4.82 35.71–38.84	23.29 ± 4.82 21.73–24.86	11.48 ± 1.86 10.55–12.41
Uric acid (mg/dL) *	6.95 ± 1.51 6.36–7.53	6.10 ± 1.49 5.62–6.58	6.42 ± 1.62 5.89–6.94	6.95 ± 2.59 5.66–8.24
Total cholesterol (mg/dL) *	172.11 ± 41.45 156.03–188.18	164.28 ± 31.00 154.23–174.33	158.46 ± 35.15 147.07–169.86	162.00 ± 37.71 143.25–180.75
HDL-c (mg/dL) *	46.79 ± 13.63 41.50–52.07	48.18 ± 10.49 44.78–51.58	44.59 ± 12.75 40.46–48.72	46.33 ± 13.94 39.40–53.27
LDL-c (mg/dL) *	107.36 ± 36.23 93.31–121.41	100.15 ± 23.16 92.64–107.66	94.67 ± 26.32 86.13–103.20	101.17 ± 31.62 85.44–116.89
Triglycerides (mg/dL) **	129.5 94.25–171.00	117.00 81.00–168.5	144.00 99.50–195.00	112.00 78.00–171.00
Total proteins (g/dL) *	6.94 ± 0.32 6.82–7.07	7.13 ± 0.41 6.99–7.26	7.04 ± 0.46 6.89–7.19	6.9 ± 0.58 6.60–7.19
Hemoglobin (g/dL) *	13.43 ± 1.27 12.94–13.93	12.50 ± 1.70 11.95–13.05	12.51 ± 1.10 12.15–12.87	11.32 ± 1.31 10.67–11.97
Glucose (mg/dL) **	112.5 100.25–144.5	112.00 95.00–136.00	116.00 100.00–142.00	101.5 91.00–135.00
Serum creatinine (mg/dL) **	1.36 1.15–1.45	1.69 1.46–1.84	2.32 2.02–2.89	4.58 4.08–5.18
Urea (mg/dL) **	56.5 46.5–67.00	72.00 58.5–82.5	93.00 86.5–112.00	144.00 126.00–179.00
Iron (µg/dL) **	67.5 52.5–76.5	67.00 51.00–91.50	75.00 60.50–92.50	60.00 45.00–67.00
TSAT (%) **	22.05 15.63–25.85	20.40 16.05–26.70	26.20 19.55–31.10	20.70 16.90–25.10
Ferritin (ng/mL) **	95.00 32.25–140.05	94.00 64–150	92.00 60.50–200.00	192.50 146.00–310.00
i-PTH (pg/mL) **	63.5 50.25–72.75	75.00 58.00–107.5	133.00 97.00–183.50	311.00 228.00–389.00
25-OH cholecalciferol (ng/mL) **	22.00 12.25–33.75	22.00 15.50–30.50	31.00 17.50–40.00	18.50 11.00–30.00
Calcium (mg/dL)**	9.76 9.52–10.17	9.60 9.30–9.95	9.60 9.30–9.85	9.20 8.90–9.50
Phosphorus (mg/dL) **	3.15 2.8–3.45	3.2 2.9–3.5	3.50 3.20–4.10	4.80 4.20–5.70

* Average ± D. Typical. 95% CI ** Median + Interquartile range.

**Table 5:** Assessment of oxidative parameters according to stage.

CKD	G3A	G3B	G4	G5-NDD	CONTROLS	*p*
8-oxo-dG (U/10^6^ dG) *	4.51 ± 0.86 (4.22–4.8)	5.88 ± 1.00 (5.59–6.18)	7.31 ± 1.09 (6.99–7.63)	7.59 ± 1.34 (7.02–8.15)	2.75 ± 1.09 (2.43–3.08)	0.001
GSH (U/mg protein) *	19.52 ± 2.73 (18.61–20.43)	18.31 ± 2.48 (17.58–19.04)	15.92 ± 2.58 (15.16–16.68)	15.50 ± 2.82 (14.30–16.69)	23.35 ± 5.26 (21.79–24.92)	0.001
GSSG (U/mg protein) *	4.02 ± 0.87 (3.73–4.32)	5.28 ± 0.93 (5.01–5.56)	6.25 ± 1.04 (5.94–6.56)	6.94 ± 1.23 (6.42–7.46)	0.33 ± 0.18 (0.28–0.38)	0.001
GSSG/GSH *	21.52 ± 8.53 (18.68–24.37)	29.58 ± 7.92 (27.25–31.91)	40.35 ± 9.75 (37.48–43.21)	46.08±10.75 (41.53–50.62)	1.33 ± 0.6 (1.15–1.51)	0.001
MDA (nmol/mg protein) *	0.57 ± 0.11 (0.53–0.61)	0.74 ± 0.12 (0.71–0.78)	0.92 ± 0.13 (0.88–0.96)	0.96 ± 0.17 (0.89–1.03)	0.11 ± 0.05 (0.93–0.12)	0.001

* Average ± D. Typical. 95% CI.

## Data Availability

All the data is available in the nephrology department at Hospital Clinico Universitario of Valencia and the Biomedical research institute of Valencia.
